# Genomic Characterization of Selected ESKAPEEE Clinical Isolates From Mbarara Regional Referral Hospital, Southwestern Uganda: Resistome, Multilocus Sequence Typing, Mobilome, and Virulome

**DOI:** 10.7759/cureus.107624

**Published:** 2026-04-23

**Authors:** Judith Owokuhaisa, Joel Bazira, Moses Mpeirwe, Godwin Tusabe, Edgar Mulogo

**Affiliations:** 1 Department of Microbiology, Mbarara University of Science and Technology, Mbarara, UGA; 2 Department of National Health Laboratory and Diagnostics Services, Central Public Health Laboratories, Ministry of Health, Kampala, UGA; 3 Department of Community Health, Mbarara University of Science and Technology, Mbarara, UGA

**Keywords:** eskapee pathogens, high-risk clones, mobile genetic elements (mges), multi locus sequencing, resistome, virulome

## Abstract

Background: The global dissemination of* Escherichia coli, Staphylococcus aureus, Klebsiella pneumoniae, Acinetobacter baumannii, Pseudomonas aeruginosa, Enterococcus faecium, Enterococcus faecalis, *and *Enterobacter cloacae* pathogens is increasingly driven by the convergence of multidrug resistance (MDR) and hypervirulence. These opportunistic pathogens are characterized by their ability to "escape" the bactericidal effects of conventional antibiotics through a refined repertoire of resistance mechanisms. Infections related to these pathogens are responsible for the majority of hospital-acquired infections (HAIs) globally, leading to increased morbidity, mortality, and healthcare costs.

Methods: This study used whole-genome sequencing to identify the resistome, virulome, mobilome, and multilocus sequence types of selected clinical *Escherichia coli, Staphylococcus aureus, Klebsiella pneumoniae, Acinetobacter baumannii, Pseudomonas aeruginosa, Enterococcus faecalis, *and *Enterobacter cloacae* pathogens.

Results: Molecular profiling revealed a diverse virulome, with *Pseudomonas aeruginosa* (ST244) exhibiting the most expansive repertoire (>130 virulence factors (VFs)), including type III secretion system effectors (*exoS, exoT*) and complete alginate biosynthesis (*alg*) clusters. *S. aureus* (ST1) demonstrated a potent toxigenic profile characterized by the Panton-Valentine Leukocidin (*lukF/S-PV*) and toxic shock syndrome toxin-1 (*tsst-1*) genes.​​​ Among the Enterobacteriaceae, virulence was dominated by the yersiniabactin (*ybt*) and enterobactin (*ent/fep*) iron-acquisition systems, which were notably conserved across *Klebsiella pneumoniae* (ST307) and *E. coli* (ST448). Furthermore, the presence of the *Escherichia coli *common pilus (*ecp*) and curli fimbriae (*csg*) across multiple species suggests that mobile genetic elements (MGEs) facilitate the horizontal transfer of "survival packages" that combine colonization and persistence traits. In addition, these isolates also revealed diverse plasmids varying significantly in size (1,570 bp to 235,562 bp) and GC content (0.28 to 0.65). Conjugative plasmids were exclusively large (>60 kb) and characterized by the presence of both a relaxase (primarily MOBH or MOBP) and a mate-pair formation (MPF) system (predominantly MPF_F and MPF_T).

Conclusion: These findings identify a critical genetic variation where high-risk bacteria combine multidrug resistance with an enhanced ability to cause disease. The emergence of these multidrug-resistant bacteria highlights an urgent need for genomic tracking in clinical settings to monitor and prevent their spread.

## Introduction

The global rise of antimicrobial resistance (AMR) has positioned the ESKAPEEE pathogens: *Enterococcus faecium*, *Staphylococcus aureus*, *Klebsiella pneumoniae*, *Acinetobacter baumannii*, *Pseudomonas aeruginosa*, *Enterobacter* spp., and *Escherichia coli*, as the primary threats to contemporary healthcare [1]. These opportunistic pathogens are characterized by their ability to "escape" the bactericidal effects of conventional antibiotics through an advanced repertoire of resistance mechanisms. Recent literature suggests that ESKAPEEE-related infections are responsible for the majority of hospital-acquired infections (HAIs) worldwide, leading to increased morbidity, mortality, and healthcare costs [1,2].

In Uganda, AMR is particularly challenging due to high infection rates and limited therapeutic options. Surveillance data, including studies from Mbarara Regional Referral Hospital (MRRH), indicate an alarming rise in multidrug-resistant (MDR) infections, with high resistance rates observed against commonly used antibiotics such as ampicillin, ciprofloxacin, and tetracycline. Recent studies at MRRH (2015-2022) revealed that over 84.1% of bacterial isolates from clinical specimens were ESKAPEEE pathogens [3]. These pathogens utilize diverse strategies, including the production of siderophores for iron sequestration (e.g., yersiniabactin), the formation of robust biofilms via curli fimbriae and adhesins, and the deployment of specialized secretion systems, such as the type III secretion system (T3SS), to inject cytotoxins directly into host cells [4]. The expression of these factors allows the bacteria to bypass the host’s innate immune response and persist in nutrient-limited clinical environments. Central to this genetic variability is the mobilome, which comprises mobile genetic elements (MGEs) such as plasmids, transposons, and insertion sequences (ISs). MGEs act as the primary vehicles for the horizontal gene transfer (HGT) of both antibiotic resistance genes (ARGs) and virulence factors (VFs) [5]. In many high-risk lineages, ARGs and VFs are physically linked on the same conjugative plasmid, meaning that antibiotic selection pressure in hospital settings inadvertently selects for more virulent strains [6]. In Ugandan clinical settings, the acquisition of MGEs has been linked to the emergence of MDR pandemic clones, such as *E. coli* ST131 and *K. pneumoniae* ST147, which often carry significant resistance determinants like blaCTX_M_15 [7].

Despite the high burden of AMR, there is limited molecular data on the specific genetic information on resistance and virulence in clinical isolates from clinical settings in Uganda. Previous studies have largely focused on phenotypic resistance testing and multiplex PCR, which provide limited information on the complete genetic resistance in the pathogens [8]. This study represents the first comprehensive molecular characterization of ARGs, virulence, MGEs, and sequence types (STs) in selected ESKAPEEE isolates within the clinical setting of MRRH in Southwestern Uganda. While global resistance trends are well documented, localized (MRRH) genomic drivers are often underinvestigated in regional surveillance. This study aims to: (1) identify the presence and diversity of ARGs conferring resistance to various antibiotic classes, (2) perform multilocus sequencing typing in line with internationally recognized high-risk clones, (3) identify and characterize MGEs such as integrons and transposons, and (4) identify virulence factors like biofilm formation, toxins, and secretion systems. This research reveals data that contribute to understanding AMR potential contributors at MRRH, Southwestern Uganda, and preliminary information on AMR in a resource-limited setting.

## Materials and methods

Bacterial strains and clinical source

We purposively selected eight isolates to represent the most clinically significant and MDR phenotypes among *Escherichia coli*, *Staphylococcus aureus*, *Klebsiella pneumoniae*, *Acinetobacter baumannii*, *Pseudomonas aeruginosa*, *Enterococcus faecium*​​,*​Enterococcus faecalis*, and *Enterobacter cloacae* pathogens currently circulating at Mbarara Regional Referral Hospital (MRRH) between July and August 2024. Since our study was an exploratory characterization of the resistome, virulome, sequence types, and mobilome (presence or absence) rather than a statistical prevalence, a small, targeted sample size was deemed to answer the study objective. Research has shown that a minimal sample of three to eight individuals can be sufficient to accurately estimate genetic diversity and identify high-risk international clones (STs) when using high-density molecular markers like whole-genome sequencing (WGS). By prioritizing isolates with confirmed extensive resistance profiles, this study maximized the probability of capturing rare or emerging resistance genes and mobile genetic elements (MGEs) relevant to the Ugandan clinical context, serving as a high-resolution pilot for future large-scale surveillance studies. These isolates were originally cultivated from diverse clinical samples, including pus, urine, vaginal swabs, and ear swabs. Institutional ethical clearances (MUST-2024-1766 and HS6636ES) and laboratory standard operating guidelines were followed during sample handling and processing.

Storage and revitalization

Isolates were maintained as long-term glycerol stocks (20% v/v) stored at -80°C to ensure genetic stability. For revitalization, the isolates were thawed on ice and sub-cultured onto nutrient-rich agar bases. Initial phenotypic identification and biochemical confirmation were conducted using the Vitek 2.0 Compact Automated System version 9.04.4 (bioMérieux, Marcy-l'Étoile, France) [9], which utilizes colorimetric reagent cards to provide species-level identification.

Culture conditions and growth media

Standardized cultivation protocols were followed to ensure optimal growth for genomic and phenotypic assays. All isolates, with the exception of *Enterococcus *species, were inoculated into Luria-Bertani (LB) broth (Difco, USA) and incubated at 37°C for 18-24 hours under aerobic conditions with constant agitation (150 rpm) to facilitate exponential growth. *Enterococcus faecalis* and *Enterococcus faecium* were cultivated in brain heart infusion (BHI) medium (Oxoid, UK), a highly nutritious infusion base required for the fastidious growth requirements of the *Enterococcaceae* family [10]. Purity checks were performed for each subculture via Gram staining and colony morphology assessment on selective media before proceeding to molecular characterization.

Bacterial DNA extraction and quantification

Bacterial isolates were subjected to high-speed centrifugation at 8,000 × g for five minutes to obtain a cell pellet. Genomic DNA (gDNA) was extracted from the resulting pellet using the QIAamp DNA Mini Kit (Qiagen, Hilden, Germany) following the manufacturer’s instructions. The concentration and integrity of the extracted DNA were determined using the Qubit 4 Fluorometer (Thermo Fisher Scientific, Waltham, MA, USA) with the dsDNA High Sensitivity (HS) Assay Kit.

Library preparation and whole-genome sequencing (WGS)

Prior to library construction, gDNA was normalized to a starting input of 15 ng. Sequencing libraries were prepared using the Illumina DNA Prep Kit (Illumina, Inc., San Diego, CA, USA). Quality control of the final libraries was performed using the DNA High Sensitivity Assay (Agilent Technologies, Santa Clara, CA, USA) to ensure optimal fragment size distribution. The libraries were subsequently sequenced on the Illumina MiSeq platform, generating 150 bp paired-end reads.

Bioinformatics analysis and genome assembly

The quality of the raw sequence data was assessed using FastQC v0.12.1 [11]. Adapter sequences and low-quality bases (Q30 score <28) were removed using Trimmomatic v0.40 [12]. De novo assembly of the high-quality reads was performed using SKESA v2.5.1 [13], a k-mer-based assembler optimized for microbial genomes.

Genomic characterization

The assembled genomes were analyzed as follows: antimicrobial resistance (AMR) was detected using ResFinder [14], Plasmid Identification through PlasmidFinder 2.1 and the plasmidfinder_db (2023-01-18) [15], multilocus sequence typing (MLST) for the sequence types (ST) was determined using DTU MLSTv1.0.1 and the pubMLST database [16], and virulence factors using VirulenceFinder 2.0.5 and the virulence finder_db (2022-12-02) [17]. Only AMR genes detected with at least 90% coverage were reported. Reads that had higher N50 and lower L50 were considered for further analysis. Post-assembly quality control was performed using QUAST v4.1 (QUality Assessment Tool for genome assemblies, Center for Algorithmic Biotechnology (CAB), St. Petersburg State University, Russia). The raw sequence reads can be accessed under Accession Number PRJNA1439330 under raw sequence research in the Sequence Read Archive (SRA).

## Results

Diversity and distribution of antimicrobial resistance genes (ARGs) in selected ESKAPEEE clinical isolates

**Figure 1:**
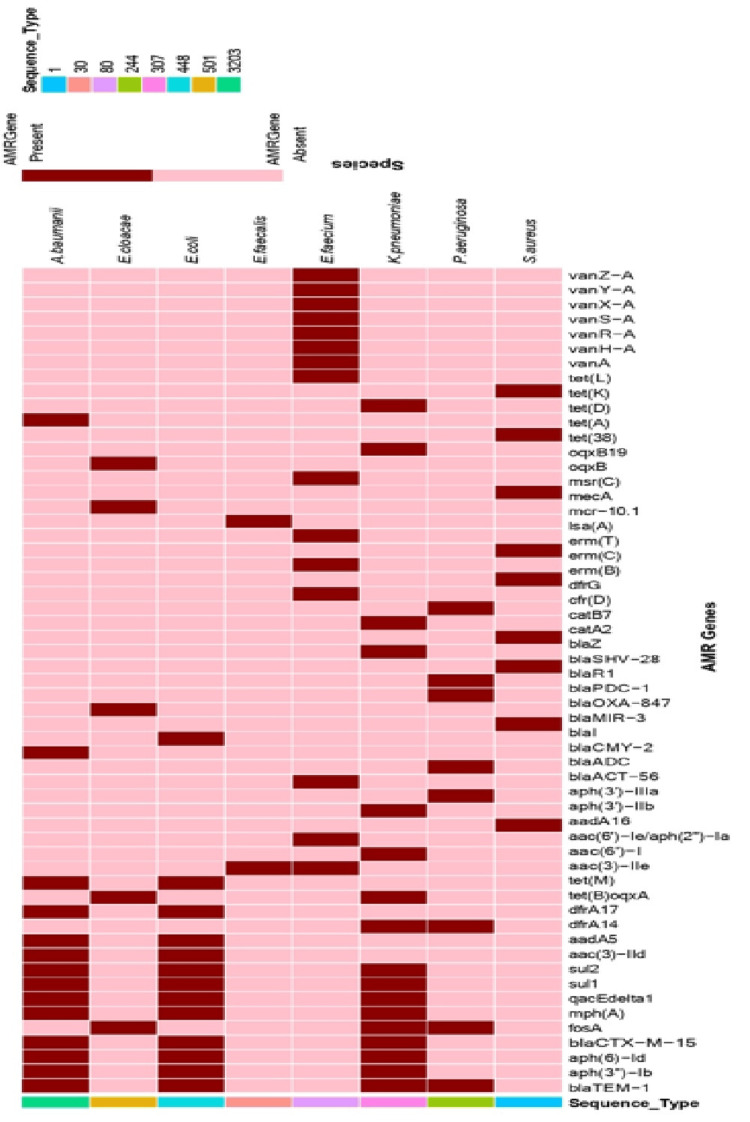
A heat map showing antimicrobial resistance gene profile of ESKAPEEE isolates. The figure shows the presence (maroon for antimicrobial resistance genes) or absence (pink) of antimicrobial resistance genes. The figure shows a detailed profile of antimicrobial resistance genes and multilocus sequence types of *Escherichia coli*, *Staphylococcus aureus*, *Klebsiella pneumoniae*, *Acinetobacter baumannii*, *Pseudomonas aeruginosa*, *Enterococcus faecium*, *Enterococcus faecalis*, and *Enterobacter cloacae.*

Genomic analysis revealed a total of 57 antimicrobial resistance genes (ARGs) in *E. coli, S. aureus, K. pneumoniae, A. baumannii, P. aeruginosa, E. faecium, E. faecalis, and E. cloacae* (ESKAPEEE) eight isolates. The genes span across 11 antibiotic classes and resistance to quaternary ammonium compounds (QACs) (Table 1, Figure 1). The distribution of these genes varied across the investigated ESKAPEEE isolates, with* K. pneumoniae* and *E. coli *displaying the most diverse resistomes.

Aminoglycoside-modifying enzymes (AMEs) were the most widely distributed class of ARGs, detected in seven out of the eight species except *E. cloacae*. The range included acetyl transferases (e.g., (3)−*IId*), adenylyl transferases (*aadA5*), and phosphotransferases (*aph(3′)*−*IIIa*). A diverse array of *β*-lactamase genes was identified. Broad-spectrum and extended-spectrum *β*-lactamases (ESBLs), such as *blaCTX*-*M*-15, *blaTEM*-1, and *blaSHV*-28, were prevalent in the Gram-negative isolates (*E. coli, K*. *pneumoniae*, and *A. baumannii*). Notably, *S. aureus* harbored the *mecA* gene, confirming its status as methicillin-resistant *S. aureus* (MRSA), alongside the regulatory genes *blaI* and *blaR*1. *K. pneumoniae, E. faecium*,and *E. faecalis* carried catB7, catA2, and cfr(D) ARGs, which confer resistance to chloramphenicol (Table 1, Figure 1).

Tetracycline resistance was mediated by efflux pumps (e.g., (*A*), *tet*(*B*)) and ribosomal protection proteins (e.g., *tet(M)*, *tet(L)*), found across both Gram-positive and Gram-negative isolates, except *P. aeruginosa* and* E. cloacae. *The majority of the isolates (7/8) showed resistance to last-line and specialized antibiotics, such as glycopeptides, except *E. faecium*.Resistance to clinical last-line antibiotics was species-specific. A van A gene cluster (*van A*, *H*, *R*, *S*, *X*, *Y*, Z) was identified exclusively in *E. faecium* related to vancomycin resistance.

Fluoroquinolone resistance via the plasmid-mediated efflux system *OqxA/B* was restricted to *K. pneumoniae* and *E. cloacae* isolates. Macrolide resistance genes (*mph(A)*, *msr(C)*) and macrolide-lincosamide-streptogramin B (MLSB) determinants (*erm(B)*, *erm(C)*, *erm(T)*) were identified in *E. coli* and *E. faecium*. Furthermore, the colistin resistance gene, *mcr*-10.1, was uniquely detected in *E. cloacae* (Table 1, Figure 1). In addition, the *fosA *gene, conferring resistance to fosfomycin, was identified in *K. pneumoniae*, *P. aeruginosa*, and *E. cloacae*.

The results of the study also reveal that some of the ESKAPEEE clinical isolates were multidrug-resistant and antiseptic-tolerant. They showed macrolides and MLSB phenotype resistance. Also, the *qacE*Δ1gene, which confers reduced susceptibility to quaternary ammonium compounds (QACs) used in hospital disinfection, was identified in *E. coli*, *K. pneumoniae*, and *A. baumannii*. This gene was consistently co-located in isolates also harboring sulfonamide resistance (*sul*1, *sul*2).

**Table 1:** Diversity and distribution of antimicrobial resistance genes (ARGs) in ESKAPEEE clinical isolates. +Represents genes present in the isolate, -Genes absent in the isolate. ARG: antimicrobial resistance genes: classified according to antimicrobial class and identified by whole-genome sequencing of the bacterial isolate, ESKAPEEE: E-*Escherichia coli, S-Staphylococcus aureus, K-Klebsiella pneumoniae, A-Acinetobacter baumannii, P-Pseudomonas aeruginosa, E-Enterococcus faecium, E-Enterococcus faecalis, E-Enterobacter cloacae.*

Antimicrobial class and ARGs	E. coli	S. aureus	K. pneumoniae	A. baumannii	P. aeruginosa	E. faecium	E. faecalis	E. cloacae
Aminoglycoside aac(3)-IId, aadA5, aph(3'')-Ib, aph(6)-Id, lsa(A), aac(6')-I, aph(3')-IIIa, aac(3)-IIe, aph(3')-IIb, aac(6')-Ie/aph(2'')-Ia, oqxB19, aadA16	+	+	+	+	+	+	+	-
Beta-lactam blaADC, blaCTX-M-15, blaTEM-1, blaMIR-3, blaCMY-2, blaSHV-28, blaACT-56, blaOXA-847, blaPDC-1, blaI, blaR1, blaZ, mecA	+	+	+	+	+	-	-	+
Chloramphenicol catB7, catA2, cfr(D)	-	-	+	-	+	+	-	-
Tetracycline tet(B), tet(A), tet(38), tet(K), tet(M), tet(L), tet(D)	+	+	+	+	-	+	+	-
Trimethoprim/sulfamethoxazole dfrA17, sul1, sul2, dfrA14, dfrG	+	+	+	+	+	-	-	-
Glycopeptide vanA, vanH-A, vanR-A, vanS-A, vanX-A, vanY-A, vanZ-A	-	-	-	-	-	+	-	-
Fluoroquinolones oqxA, oqxB	-	-	+	-	-	-	-	+
Macrolide mph(A), msr(C)	+	-	+	+	-	+	-	-
Polymyxin mcr-10.1	-	-	-	-	-	-	-	+
Macrolide-Lincosamide-Streptogramin B(MLSB) erm(C), erm(B), erm(T)	+	-	-	-	-	+	-	-
Quaternary ammonium compounds (QACs) antiseptic class qacEdelta1	+	-	+	+	-	-	-	-
Fosfomycin fosA	-	-	+	-	+	-	-	+

Multilocus sequencing typing (MLST) on ESKAPEEE isolates

We performed MLST analysis on ESKAPEEE isolates (n=8) and identified eight sequence types. The MLST revealed that the eight ESKAPEEE isolates belong to globally documented high-risk clones and rising lineages linked with multidrug resistance (MDR). The Gram-positive isolates were identified as *E. faecium ST80*, *E. faecalis ST30*, and *S. aureus ST1*. Among the Gram-negative pathogens, the Enterobacteriaceae included *K. pneumoniae ST307*, *E. coli ST448*, and *E. cloacae ST501*. The non-fermenting Gram-negative isolates were identified to be *P. aeruginosa* ST244 and *A. baumannii* ST3203. The distribution of sequence types and their associated resistance profiles is summarized in Table 2, Figure 1.

**Table 2:** MLST and resistome profiles of the ESKAPEEE isolates. ARG: antimicrobial resistance gene, ST: sequence typing, VRE: vancomycin-resistant enterococci, R: resistance, MRSA: methicillin-resistant *Staphylococcus aureus,* MDR: multidrug-resistant, ESBL: extended-spectrum beta-lactamase, MLST: multilocus sequence typing, ESKAPEEE: *Escherichia coli, Staphylococcus aureus, Klebsiella pneumoniae, Acinetobacter baumannii, Pseudomonas aeruginosa, Enterococcus faecium, Enterococcus faecalis, Enterobacter cloacae.*

Species	Sequence type (ST)	Key phenotype	Major ARGs detected
E. faecium	ST80	VRE	vanA, vanH, vanR, vanS, vanX, vanY, vanZ, erm(B), tet(M), aph(3')-IIIa
S. aureus	ST1	MRSA	mecA, blaZ, blaI, blaR1, erm(C), tet(38), tet(K), aac(6')-Ie/aph(2'')-Ia
K. pneumoniae	ST 307	MDR/ESBL	blaCTX-M-15, blaSHV-28, blaTEM-1, oqxA/B, fosA, catA2, qacEΔ1, sul1/2
A. baumannii	ST 3203	MDR	blaADC, blaOXA-847, aac(3)-IId, aph(3'')-Ib, aph(6)-Id, tet(B), sul2, qacEΔ1
P. aeruginosa	ST 244	MDR	blaPDC-1, aph(3')-IIb, catB7, fosA, sul1, dfrA17
E. cloacae	ST 501	Colistin-R	mcr-10.1, blaMIR-3, blaACT-56, oqxB, fosA, aac(6')-I, dfrA14
E. coli	ST 448	MDR/ESBL	blaCTX-M-15, blaTEM-1, blaCMY-2, mcr-1, mph(A), tet(A), dfrA17, sul1, qacEΔ1
E. faecalis	ST 30	MDR	lsa(A), tet(M), tet(L), dfrG

Characterization of the mobilome of ESKAPEEE isolates

The genomes of the ESKAPEEE isolates were characterized by a varied range of mobile genetic elements (MGEs), including plasmids, replicons, transposons, insertion sequences (IS), and miniature inverted-repeat transposable elements (Table 3). The Gram-negative isolates exhibited high plasmid complexity; *E. coli* and *A. baumannii* shared a similar plasmid profile, notably harboring IncC, IncFIB, and IncHI1A/B replicons. In* K. pneumoniae,* the mobilome comprises IncFIB(K), IncFII(K), and IncR plasmids. *E. cloacae *isolate harbored the repE(pEh60-7) replicon, while *P. aeruginosa* carried the IncFIB(pECLA) plasmid.

The Gram-positive *S. aureus* utilized a different set of replicons (rep5a, rep10, rep16, rep7c), whereas no conventional plasmid replicons were identified in *E. faecium *or *E. faecalis*. Transposon activity was specifically noted in the *Enterococci* and *Enterobacter* isolates. *E. faecalis* harbored Tn6009, while *E. cloacae* contained Tn6024, both of which are known vehicles for antibiotic resistance genes (Table 3). A group of insertion sequences was identified across all isolates. IS6100 was the most ubiquitous, appearing in* E*.* coli, K. pneumoniae, A. baumannii, and P. aeruginosa*. Species-specific IS elements were also prominent, such as ISAba1 in *A. baumannii *and the ISKpn series (ISKpn1, 8, 12, 21, 24, 26, 28) in *K. pneumoniae*. For all the isolates,* E. coli *was the only isolate to harbor miniature inverted-repeat transposable elements (MITEEc1).

**Table 3:** The mobilome of ESKAPEEE isolates. -Represents no plasmids, replicons, transposons, insertion sequences (IS), and miniature inverted-repeat transposable elements (MITEs) detected during whole-genome sequencing of *Escherichia coli, Staphylococcus aureus, Klebsiella pneumoniae, Acinetobacter baumannii, Pseudomonas aeruginosa, Enterococcus faecium, Enterococcus faecalis, Enterobacter cloacae *(ESKAPEEEE).

Bacterial isolates	Plasmids/replicons	Transposons	Insertion sequences	MITEs
E. coli	Col(BS512), IncC, IncFIA, IncFIA(HI1), IncFIB(AP001918), IncHI1A, IncHI1B(R27), IncI2	_	IS6100 ISEc38 ISKpn8 ISEc9 IS5 ISEc1	MITEEc1
S. aureus	rep 5a, rep 10, rep 16, rep 7c	_	_	_
K. pneumoniae	IncFIB(K), IncFIB(pNDM-Mar), IncFII(K), IncR, RepB	_	ISKpn28 ISKpn12 ISKpn21 ISEc9 ISKpn24 ISKpn26 ISKpn1 IS6100 IS5075 ISEcl1	_
A. baumannii	Col(BS512), IncC, IncFIA, IncFIA(HI1), IncFIB(AP001918), IncFII(pAMA1167-NDM-5), IncHI1A, IncHI1B(R27), IncI2	_	IS6100 ISAba1 ISEc31 ISKpn8 ISEc38 ISEc45 IS5075 IS421 ISAba33	_
P. aeruginosa	IncFIB(pECLA)	_	IS6100 ISPst2 IS5 ISPst4 ISPa22 ISPa32 ISPa6 ISPa1 ISSen4	_
E. faecium	_	_	ISEnfa4 IS16 ISSsu5	_
E. faecalis	_	Tn6009	IS1062 ISLgar5 ISS1N cn_11768_ISLgar5	_
E. cloacae	repE(pEh60-7)	Tn6024	IS26 ISKpn42 IS30 IS102	_

Detailed plasmid analysis of eight ESKAPEEE isolates also revealed a diverse picture of plasmids varying significantly in size (1,570 bp to 235,562 bp) and GC content (0.28 to 0.65). Gram-negative isolates exhibited higher plasmid complexity compared to Gram-positive isolates. Specifically,* K. pneumoniae* and* E. coli *isolates harbored the largest plasmids, with *K. pneumoniae* AA405 reaching 235,562 bp. For mobility profiles and transfer machinery of the plasmids, they were (plasmids) categorized based on their predicted mobility into conjugative, mobilizable, and non-mobilizable groups. Conjugative plasmids were exclusively large (>60 kb) and characterized by the presence of both a relaxase (primarily MOBH or MOBP) and a mate-pair formation (MPF) system (predominantly MPF_F and MPF_T). Mobilizable plasmids, such as *E. coli *AA579 and Pseudomonas AA531, typically lacked MPF systems but possessed relaxases (e.g., MOBQ, MOBV) or origin of transfer (oriT) sequences. Non-mobilizable plasmids were frequent among smaller plasmids (<5 kb) and in Pseudomonas AE985 (174.9 kb) (Table 4).

Study findings revealed that high-risk resistance clusters, including blaCTX-M-15, sul1, and aac(3)-IId, were found on large IncF-type and IncC plasmids in *E. coli, A. baumannii, *and *K. pneumoniae*. In Gram-positive isolates, resistance was linked to specific replicons; for instance, VanHAX (vancomycin resistance) was identified on a 114.7 kb non-mobilizable plasmid in *E.* *faecium*, while erm(C) and erm(T) were found on smaller mobilizable or non-mobilizable elements (Table 4).

**Table 4:** Detailed genomic and mobility profiles of plasmids in ESKAPEEE isolates. ID: identification, AMR: antimicrobial resistance, bp: base pair, GC: genome content, ESKAPEEE-*Escherichia coli*, *Staphylococcus aureus*, *Klebsiella pneumoniae*, *Acinetobacter baumannii*, *Pseudomonas aeruginosa*, *Enterococcus faecium*, *Enterococcus faecalis*, *Enterobacter cloacae*, *novel_e542628129bb2a3338c17a1a7af79ffb, *novel_bdd63cdede3071f6bea9ca0a77ee25fe, ^a^Relaxase/MPF/OriT-indicates relaxase/mating pair formation/origin of transfer, -/-/- indicates no detection of relaxase/mating pair formation/origin of transfer, + indicates additional genes present, - indicates no antimicrobial resistance gene detected.

Organism	Plasmid ID	Size (bp)	GC	Replicon type(s)	AMR genes	Relaxase/MPF/OriT^a^	Mobility
A. baumannii	AB410	74621	0.41	rep_cluster_1656	-	-/-/-	Non-mobilizable
	AA372	64765	0.43	-	-	MOBP/MPF_T/-	Conjugative
	AA172	82904	0.52	IncFIA/FIB/FII/Q1	blaCTX-M-15 cluster	MOBF/-/-	Mobilizable
	AA998	156192	0.46	IncFIA	-	MOBH/MPF_F/-	Conjugative
	AA626	92949	0.49	IncC	-	MOBH/MPF_F/MOBH,P	Conjugative
	AE322	21660	0.52	-	-	-/-/-	Non-mobilizable
	AA579	4064	0.50	rep_cluster_1778	-	MOBQ/-/MOBQ	Mobilizable
	AE688	2977	0.34	rep_cluster_1364	-	-/-/-	Non-mobilizable
	AC748	2101	0.47	Col(BS512)	-	-/-/-	Non-mobilizable
E. cloacae	novel	49234	0.53	rep_cluster_2268	-	-/-/-	Non-mobilizable
	AA517	6943	0.56	rep_cluster_2335	-	MOBQ/-/MOBQ	Mobilizable
	AA519	3115	0.52	ColRNAI_rep_1987	-	-/-/MOB_unk	Mobilizable
E. coli	AA998	181261	0.47	IncFIA/Q1	*blaTEM-1B, tet(B)*+	MOBH/MPF_F/-	Conjugative
	AA626	90687	0.49	IncC	-	MOBH/MPF_F/MOBH	Conjugative
	AA372	64439	0.43	IncI2	-	MOBP/MPF_T/-	Conjugative
	AA172	70783	0.51	IncFIA/FIB/FII	*blaCTX-M-15, sul1*+	MOBF/-/-	Mobilizable
	AA579	4064	0.50	rep_cluster_1778	-	MOBQ/-/MOBQ	Mobilizable
	AC748	2101	0.47	Col(BS512)	-	-/-/-	Non-mobilizable
E. faecalis	AB528	66591	0.33	rep_cluster_180	-	MOBP/MPF_T/-	Conjugative
	AE310	36590	0.35	rep_cluster_992	-	MOBC/-/-	Mobilizable
E. faecium	AB369	114737	0.34	-	VanHAX	-/-/-	Non-mobilizable
	AC727	46290	0.36	rep_cluster_1968	-	-/-/-	Non-mobilizable
	AH273	27918	0.39	-	-	-/-/-	Non-mobilizable
	AC732	99659	0.35	rep_cluster_893	-	-/-/-	Non-mobilizable
	AB756	32721	0.37	rep_1093,rep_185	aph(3')-III	-/-/-	Non-mobilizable
	AA893	5670	0.36	rep_cluster_1197	-	MOBP/-/-	Mobilizable
	AC670	4783	0.34	rep_cluster_1018	tet(L)	MOBV/-/-	Mobilizable
	AC630	1570	0.29	-	erm(T)	-/-/-	Non-mobilizable
K. pneumoniae	AA405	235562	0.46	IncFIB	*blaCTX-M-15, sul1*+	MOBH/MPF_F/-	Conjugative
	AA275	221739	0.52	IncFIB/FII/R	*sul2, aph(3'')-Ib*+	MOBF/MPF_F/-	Conjugative
	AA122	4268	0.44	rep_cluster_2335	-	-/-/-	Non-mobilizable
	AB056	3983	0.44	rep_2327/2358	-	-/-/-	Non-mobilizable
P. aeruginosa	*novel	3830	0.56	rep_cluster_2392	-	MOBP/-/MOBP	Mobilizable
	*novel	12804	0.50	IncFIB	-	-/-/-	Non-mobilizable
	AE985	174960	0.65	-	-	-/-/-	Non-mobilizable
	AF876	56570	0.60	-	-	-/MPF_G/-	Non-mobilizable
	AA531	3223	0.56	ColRNAI_rep_1987	-	MOBP/-/MOB_unk	Mobilizable
	AB040	2395	0.51	rep_cluster_2358	-	-/-/MOB_unk	Mobilizable
S. aureus	AA411	20654	0.28	rep_1733/2214	-	MOBV/-/MOBQ	Mobilizable
	AC333	6914	0.30	rep_1118/1947	erm(C)	MOBV/-/-	Mobilizable

Characterization of the ESKAPEEE virulome

The study also screened the eight selected ESKAPEEE clinical isolates for virulence factors. The results revealed a diverse and species-specific collection of virulence factors (VFs) (Table 5), categorized into four main functional groups: adhesion/biofilm formation, iron acquisition (siderophores), secretory toxins, and immune evasion (Table 5). Results from the virulome analysis revealed that *Pseudomonas aeruginosa* (ST244) exhibited the most expansive virulome, harboring over 130 VFs (Table 5). This included the complete alginate biosynthesis (alg) and flagellar/pili (flg, fli, pil) systems. Notably, it carried the type III secretion system (T3SS) effectors exoS, exoT, and exoY, alongside a quorum sensing apparatus (las, rhl).

For the case of *Staphylococcus aureus* (ST1), it showed a potent toxigenic profile, including the Panton-Valentine Leukocidin (PVL) genes (lukF/S-PV), toxic shock syndrome toxin-1 (tsst-1), *Staphylococcal* enterotoxins (sea, sec, selk-sell), and the *ica* operon (biofilm) and *cap*8 (capsule) clusters (Table 4). *Escherichia coli* (ST448) was dominated by the yersiniabactin (ybt) and enterobactin (ent/fep) Iron acquisition clusters and characterized by a high density of adhesins, specifically the Afa/Dr family (afaB-I, afaC-I, draA-P) and curli fimbriae (csg). Additionally, *Enterococcus faecalis* (ST30) and *E. faecium* (ST80) isolates demonstrated particular colonization factors. *E. faecalis* harbored the cytolysin (cyl) operon and the endocarditis-associated pili (*ebp*) cluster, while *E. faecium* was characterized by the *acm* and *adsA* adhesins. 

*Klebsiella pneumoniae *(ST307) and *A. baumannii *(ST3203) had profoundly metabolic/uptake systems, including the ybt and ecp (*E. coli* common pilus) operons, and the global regulator ompA. Particularly, the Enterobacter cloacae (ST501) isolate presents a more or less virulence profile compared to the extensive virulomes of *P. aeruginosa* or *S. aureus*. It possessed Outer Membrane Protein A (O) and curli fiber secretion unit (*csgG*) and an enterobactin biosynthesis (*entA*) (Tables 5, 6), and unlike *S. aureus *or *P. aeruginosa*, this isolate lacked exotoxins.

**Table 5:** The virulome profiles of ESKAPEEE clinical isolates. Table highlights detailed virulence factors detected from each isolate of *Escherichia coli, Staphylococcus aureus, Klebsiella pneumoniae, Acinetobacter baumannii, Pseudomonas aeruginosa, Enterococcus faecium, Enterococcus faecalis, *and *Enterobacter cloacae*.

Isolate	Representative virulence factor
E. coli	afaB-I, afaC-I, csgB, csgD, csgF,csgG, daaF, draA, draD, draE2, draP, entA, entB, entC, entD, entE, entF, entS, espL1, espR1, espX1, espX4, espX5, fdeC, fepA, fepB, fepC, fepD, fepG, fes, fimA, fimB, fimC, fimD, fimE, fimF, fimG, fimH, fimI, fyuA, gspC, gspD, gspE, gspF, gspG, gspH, gspI, gspJ, gspK, gspL, gspM, irp1, irp2, ompA, yagV/ecpE, yagW/ecpD, yagX/ecpC, yagY/ecpB, yagZ/ecpA, ybtA, ybtE, ybtP, ybtQ, ybtS, ybtT, ybtU, ybtX, ykgK/ecpR
S. aureus	tsst-1, vWbp, spa, srtB, sspC, tsst-1, vWbp, sak, sbi, scn, sdrC, sdrD, sdrE, sea, sec, she, selk, sell, selq, isdA, isdB, isdC, isdD, isdE, isdF, isdG, lip, lukF-PV, lukS-PV, hlb, hld, hlgA, hlgB, hlgC, hysA, icaA, icaB, icaC, icaD, icaR, fnbA, fnbB, geh, esaA, esaB, esaC, essA, essB, essC, esxA, esxB, clfA, clfB, coa, ebp, aur, cap8A, cap8B, cap8C, cap8D, cap8E, cap8F, cap8G, cap8H, cap8I, cap8J, cap8K, cap8L, cap8M, cap8N, cap8O, cap8P, clfA, adsA
K. pnuemoniae	yagV/ecpE, yagW/ecpD, yagX/ecpC, yagY/ecpB, yagZ/ecpA, ybtA, ybtE, ybtP, ybtQ, ybtS, ybtT, ybtU, ybtX, ykgK/ecpR, fyuA, entA, entB
A. baumannii	yagZ/ecpA, ybtE, gspF, gspH, gspI, gspJ, gspL, gspM, gspC, csgF, daaF, draA, draP, afaB-I, afaC-I
E. cloacae	ompA, entA, csgG
E. faecium	sgrA, fss3, acm
E. faecalis	sprE, srtC, fsrC, fss1, fss2, fss3, gelE, EF3023, ebpA, ebpB, ebpC, efaA, cpsA, cpsB, cpsC, cpsD, cpsE, cpsG, cpsH, cpsI, cpsJ, cpsK, cylA, cylB, cylI, cylL, cylM, cylR1, cylR2, cylS, bopD
P. aeruginosa	xcpT, xcpU, xcpV, xcpW, xcpX, xcpY, xcpZ, tagF/pppB, tagQ, tagR, tagS, tagT, toxA, tse1, tse2, tse3, vgrG1a, waaA, waaC, waaF, waaG, waaP, wzy, wzz, xcpA/pilD, xcpP, xcpQ, xcpR, xcpS, pscT, pscU, ptxR, pvcA, pvcB, pvcC, pvcD, pvdA, pvdE, pvdF, pvdG, pvdH, pvdJ, pvdL, pvdM, pvdN, pvdO, pvdP, pvdQ, pvdS, rhlA, rhlB, rhlC, rhlI, pilO, pilQ, pilR, pilS, pilT, pilU, pilV, pilW, pilX, pilY1, pilY2, plcH, popD, popN, popB, ppkA, pppA, pscB, pscC, pscD, pscE, pscF, pscG, pscH, pscI, pscJ, pscK, pscL, scN, pscO, pscP, pscQ, pscR, pscS, pchA, pchB, pchC, pchD, pchE, pchF, pchG, pchH, pchI, pchR, pcr1, pcr2, pcr3, pcr4, pcrD, pcrG, pcrH, pcrR, pcrV, phzB1, phzG1, phzM, phzS, pilB, pilC, pilE, pilF, pilG, pilH, pilI, pilJ, pilK, pilM, pilN, lasA, lasB, lasI, lip1, mbtHlike, motA, motB, motC, motD, motY, mucA, mucB, mucC, mucD, mucE, mucP, ompA, hcp1, hsiA1, hsiB1/vipA, hsiC1/vipB, hsiE1, hsiF1, hsiG1, hsiH1, hsiJ1, icmF1/tssM1, flgK, flgL, flgM, flgN, flhA, flhB, flhF, fliA, fliC, fliD, fliE, fliF, fliG, fliH, fliJ, fliK, fliL, fliM, fliN, fliO, fliQ, fliS, fptA, fpvA, fha1, fimT, fimU, fimV, fleI/flag, fleN, fleP, fleQ, fleR, fleS, flgA, flgB, flgC, flgD, flgE, flgF, flgG, flgH, flgI, flgJ, exoS, exoT, exoY, exsA, exsB, exsC, exsD, exsE, clpV1, csgG, dotU1, algQ, alg, algU, algW, algX, algZ, aprA, chpA, chpB, chpC, chpD, chpE, alg44, alg8, algA, algB, algC, algD, algE, algF, algG, algI, algJ, algK, algL, algP/algR3

**Table 6:** Summary of virulence factor categorization by isolate. - Indicates no virulence factor under that category of virulence factors. The table categorizes the representative virulence factors into adhesion and biofilm, immune evasion, iron acquisition (siderophore) and metabolism, secretion systems, toxins, and enzymes detected in: *​​​​Escherichia coli, Staphylococcus aureus, Klebsiella pneumoniae, Acinetobacter baumannii, Pseudomonas aeruginosa, Enterococcus faecium, Enterococcus faecalis, *and *Enterobacter cloacae. *

Isolate	Adhesion and biofilm	Immune evasion	Iron acquisition (siderophore) and metabolism	Secretion systems	Toxins and enzymes
E. coli	afaB-I, afaC-I, csgB/D/F/G, daaF, draA/D/E2/P, fimA-I, ompA, fdeC, yagV-Z (ecpA-E)	espL1, espR1, espX1/4/5	entA-S, fepA-G, fes, fyuA, irp1/2, ybtA-X	*gspC-M* (T2SS)	ykgK (ecpR)
S. aureus	spa, srtB, sdrC/D/E, icaA-R, fnbA/B, clfA/B, ebp, coa	sak, sbi, scn, cap8A-P, adsA	isdA-G	*esaA-C, essA-C, esxA/B* (T7SS)	tsst-1, vWbp, sea/c/h/k/l/q, lip, lukF/S-PV, hlb/d, hlgA-C, hysA, geh, aur
K. pneumoniae	yagV-Z (ecpA-E), fimH	wcaG	ybtA-X, fyuA, entA/B	_	_
A. baumannii	yagZ (ecpA), csgF, draA/P, afaB-I, afaC-I	_	ybtE	*gspF-M* (T2SS)	_
E. cloacae	csgG	­_	entA	_	ompA
E. faecium	sgrA, fss3, acm	_	_	_	_
E. faecalis	ebpA-C, efaA, fss1-3	cpsA-K	_	srtC	sprE, fsrC, gelE, cylA/B/I/L/M/R/S, bopD
P. aeruginosa	pilB-Y, fimT/U/V, flgA-N, flhA/B/F, fliA-S, fleI-S, motA-Y, waaA-P, wzy, wzz, algA-Z	mucA-E	pvdA-S, pchA-I, fptA, fpvA, mbtH-like	*xcpA-Z* (T2SS), *pscB-U* (T3SS), *pcr1-V* (T3SS), *hcp1, hsiA-J* (T6SS), *clpV1, dotU1, vgrG1a, tse1-3*	toxA, plcH, lasA/B/I, aprA, exoS/T/Y

## Discussion

The MLST analysis underscores the clinical gravity of the isolates studied, as several belong to "high-risk international clones" known for their superior ability to colonize host tissues, evade immune responses, and disseminate resistance determinants. A critical finding in this study is the identification of *K. pneumoniae* ST307. Recent genomic surveillance indicates that ST307 is rapidly displacing other clones like ST258 in several geographical regions (Europe and the Americas). Its success is attributed to a highly conserved plasmid (IncFIIk), which provides a fitness advantage under antibiotic pressure [7,18] and is highly adapted to the hospital environment due to specific mutations in its core genome that enhance its fitness and persistence on clinical surfaces [18]. ST307 has a specific plasmid-encoded *blaCTX-M*−15 and often carries additional virulence factors (like siderophores) that make it more dangerous than standard *K. pneumoniae*. Similarly, *P. aeruginosa* ST244 is recognized as a global multidrug-resistant clone. Its role in the dissemination of metallo-*β*-lactamases (MBLs) is well documented, making it a significant driver of carbapenem resistance in healthcare settings [19,20]. The detection of* E. faecium* ST 80 is representative of the hospital-acquired (HA) Clade A1. This ST is frequently associated with the *vanA *cluster identified in our results, highlighting its role as a primary agent of vancomycin-resistant enterococci (VRE) outbreaks [21,22]. Additionally, ST 80 belongs to clonal complex 17 (CC17), which is the primary lineage responsible for the global spread of hospital-adapted, ampicillin-resistant, and vancomycin-resistant enterococci.

Conversely, *S. aureus* ST1 is a lineage of high plasticity. Although it is a well-known community-acquired (CA-MRSA) lineage, its presence in this clinical panel suggests the ongoing link between community- and hospital-acquired MRSA, often carrying high virulence loads [23]. While ST1 is often community-associated, its presence with mecA and multiple MGEs (aac(6')-Ie/aph(2'')-Ia) indicates a highly resistant hospital-adapted variant. Whereas *E. coli* ST131 is often the focus of MDR studies, the identification of *E. coli* ST448 in our study is notable. ST448 has been increasingly associated with the global spread of NDM-type carbapenemases and *mcr*genes, representing an emerging threat in the "post-antibiotic" era [24]. Furthermore, the presence of *E. cloacae* ST501 in our study is significant, as this specific lineage has been implicated in outbreaks of carbapenem-resistant *Enterobacter *spp. across multiple continents, often carrying the *blaOXA*−48 or *mcr* variants. Finding *mcr*−10.1 in ST 501 is rare and suggests that ST 501 may be becoming a significant vector for plasmid-mediated colistin resistance [25]. Moreover, ST 501 has been associated with outbreaks, suggesting that *Enterobacter* species are utilizing specific STs as vehicles for *mcr*−10.1 and other plasmid-borne resistance determinants [25]. However, some researchers revealed ST 501 carrying mcr-10 [26]. These findings illustrate that our isolates are not isolated local occurrences but are part of a broader, interconnected global network of MDR transmission, necessitating coordinated international genomic surveillance.

More still, the high-level multidrug resistance observed in our ESKAPEEE isolates is a direct result of the physical linkage between critical antimicrobial resistance genes (ARGs) and the identified mobile genetic elements (MGEs). The mobilome identified in our ESKAPEEE isolates provides the structural basis for the observed multidrug-resistant (MDR) phenotypes. The co-existence of diverse plasmid replicons and insertion sequences suggests a high genomic environment that facilitates both vertical and horizontal gene transfer (HGT). The distribution of mobile genetic elements (MGEs) and sequence types (STs) identified in this study aligns with several international surveillance reports, yet reveals unique local characteristics. A particularly significant finding in our study is the detection of *mcr*−10.1 in *E. cloacae *ST501. While the *mcr*−1 variant remains the most frequently reported colistin resistance determinant in *E. coli*, *mcr*−10.1 appears to be a specialized feature of the *Enterobacter *genus. Our results mirror recent genomic reports from Southeast Asia and Africa, where *mcr*−10.1 was localized on *repE*-type plasmids [27]. The high frequency of *IS*26 in our *E. cloacae* isolate further suggests an environment primed for the rapid reorganization of these resistance islands, a feature also noted in recent Brazilian outbreaks of ST501. Also, our study revealed the co-occurrence of the *mcr*−10.1 gene and the *r*(*pEh*60−7) plasmid replicon, which strongly suggests a plasmid-mediated mechanism for colistin resistance. This specific replicon has been increasingly identified as a stable genetic backbone for *mcr* variants, facilitating the rapid horizontal transfer of polymyxin resistance within the *Enterobacteriaceae* [27].

Similarly, the identification of IncFIB and IncR plasmids among others in* K. pneumoniae* ST307 likely provides the genetic environment for the maintenance of *blaCTX-M*−15 and *blaSHV*−28. This reflects a growing global trend where ST307 is emerging as a more resilient successor to the historically dominant ST258 lineage. Comparative studies in Europe and South America have similarly noted that the chance of ST307 is largely attributed to these stable multi-replicon genetic backbones, which facilitate the maintenance of the *blaCTX-M*−15 gene [18,27]. Insertion sequences play a dual role as both promoters of gene expression and facilitators of gene movement. The role of insertion sequences (IS) in modulating resistance phenotypes is most evident in the Gram-negative non-fermenters. In *A. baumannii* ST3203, the presence of ISAba1 upstream of the *blaADC* and *blaOXA*−847 genes is a classic mechanism for the overexpression of these *β*-lactamases, leading to high-level carbapenem resistance, similar to other literature [28]. Furthermore, the occurrence of IS6100 across *E. coli, K. pneumoniae*, and *P. aeruginosa* indicates its role as a key mediator for the mobilization of aminoglycoside and sulfonamide resistance islands (aac, aadA, sul1) [29]. 

Also, detection of MITEEc1 in *E. coli* ST448 adds to the relatively sparse data on miniature inverted-repeat transposable elements (MITEs) in clinical ESKAPEEE isolates. While most studies focus on larger insertion sequences like *IS*6100, the presence of MITEs represents a cryptic yet powerful mechanism for genomic remodeling. Previous studies have suggested that MITEs may serve as hotspots for the integration of other transposable elements, potentially explaining the high plasmid complexity (IncC, IncFIA/B) observed in our *E. coli* isolate [30]. In Gram-positive isolates, the genomic stability of resistance determinants is achieved through different mechanisms. While *S. aureus* ST1 relies on diverse rep plasmids for the carriage of *mecA* and *blaZ* (Figure 1), the *Enterococci* show a preference for transposon-mediated movement. Moreover, Gram-positive isolates showed fewer plasmid types; the existence of Tn6009 in *E. faecalis* and Tn6024 in *E. cloacae* highlights the importance of transposon-mediated resistance. Tn6009 is a well-known carrier of the tet(M) gene and is often associated with the global dissemination of tetracycline resistance in *Enterococci*. However, another study reported a novel non-composite conjugative transposon, Tn6009, with a Tn916 element linked to an *S. aureus* mer operon carrying genes coding for inorganic mercury resistance (merA), organic mercury resistance (merB), a regulatory protein (merR), and a mercury transporter (merT). This transposon was found in both Gram-positive and Gram-negative isolates and is the first transposon in the Tn916 family linked to Gram-positive mer genes, directly connecting to the tet(M) gene [31].

The lack of identifiable plasmids in* E. faecium*, despite its high resistance profile, suggests that its resistance determinants are likely integrated into the chromosome via ISEnfa4 or IS16, providing a stable reservoir for vancomycin resistance that can be vertically inherited across the CC17 lineage [32]. The presence of *IS*16 in *E. faecium* ST80 confirms its membership in the hospital-adapted CC17 lineage. This result is consistent with a large-scale meta-analysis of VRE isolates, which identified *IS*16 as the most reliable molecular marker for distinguishing clinical *E. faecium* from commensal strains [33]. Comparing the plasmid heavy profile of *S. aureus*ST1 in our study, the chromosomal stability provided by *IS*16 in *E. faecium* may explain its superior persistence in the hospital environment despite rigorous infection control measures. The convergence of hypervirulence and multidrug resistance (MDR) within specific "high-risk" clones defines the trait of current ESKAPEE pathogens, which is the case with our ESKAPEE isolates. Findings from our study continue to illustrate that these isolates have transitioned from being merely resistant to becoming highly tough and aggressive pathogens through the acquisition of integrated genetic transformations.

The identification of *K. pneumoniae *ST307 and *E. coli* ST448 in our study highlights the global shift toward "convergent" lineages. ST307 is a well-documented high-risk clone that often serves as a reservoir for carbapenemases (e.g., blaKPC and blaOXA-48) [34,35]. Our results show that this lineage also carries the yersiniabactin (ybt) and *E. coli* common pilus (ecp) clusters. Recent studies indicate that these VFs are frequently co-located with ARGs on IncFII or IncL/M plasmids, allowing for the simultaneous transmission of resistance and virulence [34]. This genetic linkage ensures that antibiotic selection pressure indirectly maintains the pathogen’s ability to sequester iron and adhere to host tissues. In *S. aureus* ST1, the presence of tsst-1 and PVL genes is inextricably linked to specific MGEs. tsst-1 is typically carried on staphylococcal pathogenicity islands (SaPIs), while it is disseminated via lysogenic bacteriophages to ensure host-to-host transmission [36]. Similarly, in *E. faecalis* ST30, the cyl (cytolysin) operon is often found on pheromone-responsive plasmids, which utilize highly efficient conjugation systems to spread both virulence and vancomycin resistance (vanA/B) across *enterococcal* populations [37]. The expansive virulome of *P. aeruginosa* ST244 (130+ VFs) and the minimalist profile of *A. baumannii* ST3203 represent two ends of the perseverance spectrum. ST244 is a globally emerging lineage frequently associated with metallo-lactamases (MBLs) like blaVIM or blaNDM. The T3SS effectors (exoS, exoT) identified in this isolate are often regulated in tandem with efflux pump systems (e.g., MexAB-OprM), creating a phenotype that is both highly cytotoxic and intrinsically resistant [38]. In *A. baumannii*, the reliance on ompA and ecp is often complemented by the AbaR-type genomic islands, which can harbor up to 40 different ARGs. These genomic islands act as "resistance anchors," allowing the ST3203 lineage to persist on hospital surfaces despite the lack of aggressive exotoxins [39].

In addition, the presence of curli fimbriae (csg) in *E. coli* ST448 and* E. cloacae* ST501 is significant in the context of the "mobilome." Curli fibers facilitate the formation of dense biofilm matrices, which act as horizontal gene transfer (HGT) "hot spots." Within these biofilms, the proximity of cells promotes the exchange of insertion sequences (IS) like IS26 and IS6100, which are known to mobilize ARGs between different species of *Enterobacteriaceae* [40]. This may be due to shared virulence traits, such as entA and ompA, across the *E. coli, K. pneumoniae*, and *E. cloacae* isolates observed in this study. Our results further demonstrate that the dissemination of multidrug resistance (MDR) in ESKAPEEE pathogens might be heavily reliant on large, self-transmissible conjugative plasmids. The correlation between plasmid size and the presence of MPF_F and MPF_T systems suggests that these mega-plasmids serve as the primary vehicles for horizontal gene transfer (HGT) in clinical environments [41]. The identification of identical AMR clusters (e.g., blaCTX-M-15, tet(B), aph(6)-Id) across different species, such as *A. baumannii *AA172 and *E. coli* AA172, highlights the role of IncF and IncC scaffolds as universal resistance platforms [42]. The presence of IS6100 and other insertion sequences, as noted in the MGE analysis in our study, likely facilitates the movement of these genes between the chromosome and diverse plasmid backbones [43]. In Gram-positive isolates like *E. faecium *ST80, the presence of VanHAX on large non-mobilizable plasmids suggests a strategy of vertical transmission rather than rapid horizontal spread, although these elements remain susceptible to mobilization in the presence of co-resident conjugative machinery [44]. The high virulome complexity of*P. aeruginosa *ST244 and the toxigenic profile of *S. aureus* ST1, combined with their respective plasmid burdens, highlight the potential changes in these pathogens' relation to pathogenicity [38]. This necessitates genomic-based surveillance for effective infection control of these resistant pathogens.

Study strengths

The study demonstrates several notable strengths. First, it employs whole-genome sequencing (WGS), which provides a comprehensive and high-resolution characterization of antimicrobial resistance, virulence factors, and mobile genetic elements. The integration of resistome, mobilome, virulome, and MLST analysis is particularly valuable and allows for a multidimensional understanding of ESKAPEEE pathogens. Additionally, the study contributes important regional data from Uganda, addressing a recognized gap in genomic surveillance in low-resource settings.

Study limitations

While this study provides information on the selected ESKAPEEE resistome, MLST, mobilome, and virulence factors, limitations must be acknowledged; first, the genomic analysis was primarily conducted using short-read sequencing (Illumina) and short-read data often result in fragmented assemblies that make it difficult to definitively localize genes to specific plasmids or chromosomal locations. Future studies utilizing long-read sequencing (e.g., Oxford Nanopore) would be beneficial to fully capture the structure of the mobile genetic elements carrying the *mcr*−10.1 and *vanA* clusters and to validate their functionality, location, and gene linkage.

Secondly, the very small sample size (n=8), which substantially restricts the generalizability of the findings, and the study largely being descriptive, lacking statistical analysis, comparative genomics, or phylogenetic context, limit the depth of interpretation. However, the study focused on detailed characterization rather than statistical prevalence. The samples were not randomly chosen but purposively selected to represent specific phenotypes. So, by selecting eight isolates with well-documented specific phenotype resistance patterns, the study focused on the genetic determinants of those specific traits, bridging the gap (at the study setting) to link phenotype to genotype. Additionally, WGS produces a vast amount of data per sample, so eight samples could provide ample data for identifying resistance genes, virulence factors, or plasmid profiles without needing many samples. Many base pairs per bacterial genome are produced for every single isolate. Also, we aimed to identify how a bacterium is resistant, not to calculate the prevalence of that resistance in the population. Eight samples were deemed to provide enough variation in resistance in the study setting. Therefore, we recommend prevalence-related studies in the population involving larger sample sizes, diverse clinical and geographical settings, and comparative genomic analysis.

Finally, the isolates analyzed represent a specific geographical setting (MRRH-Southwestern Uganda); therefore, the findings of these specific clones and genes may not reflect the full diversity of ESKAPEEE resistance in other regions or clinical settings. This creates a need for surveillance of ESKAPEEE pathogens from diverse regions and clinical settings.

## Conclusions

This study reveals that eight purposively selected clinical ESKAPEEE isolates from MRRH carried high-risk clones (ST307, ST244, ST1), as well as the vanA and mcr-10.1 genes. Finding both large plasmids (>60 kb) and smaller plasmids carrying resistance genes necessitates clinical setting safety plans to focus more on tracking plasmids that carry resistance in routine antimicrobial resistance surveillance.
